# Novel Strategies for Nanoparticle-Based Radiosensitization in Glioblastoma

**DOI:** 10.3390/ijms22189673

**Published:** 2021-09-07

**Authors:** Henry Ruiz-Garcia, Cristopher Ramirez-Loera, Timothy D. Malouff, Danushka S. Seneviratne, Joshua D. Palmer, Daniel M. Trifiletti

**Affiliations:** 1 Department of Radiation Oncology, Mayo Clinic, Jacksonville, FL 32224, USA; ruizgarcia.henry@mayo.edu (H.R.-G.); Malouff.timothy@mayo.edu (T.D.M.); Seneviratne.Danushka@mayo.edu (D.S.S.); 2 Department of Neurological Surgery, Mayo Clinic, Jacksonville, FL 32224, USA; cvrl_69@live.com.mx; 3 Department of Radiation Oncology, Ohio State University, Columbus, OH 43210, USA; Joshua.palmer@osumc.edu

**Keywords:** nanoparticle, radiotherapy, radiosensitization, glioblastoma, stem-cells, theranostic

## Abstract

Radiotherapy (RT) is one of the cornerstones in the current treatment paradigm for glioblastoma (GBM). However, little has changed in the management of GBM since the establishment of the current protocol in 2005, and the prognosis remains grim. Radioresistance is one of the hallmarks for treatment failure, and different therapeutic strategies are aimed at overcoming it. Among these strategies, nanomedicine has advantages over conventional tumor therapeutics, including improvements in drug delivery and enhanced antitumor properties. Radiosensitizing strategies using nanoparticles (NP) are actively under study and hold promise to improve the treatment response. We aim to describe the basis of nanomedicine for GBM treatment, current evidence in radiosensitization efforts using nanoparticles, and novel strategies, such as preoperative radiation, that could be synergized with nanoradiosensitizers.

## 1. Introduction

Glioblastoma (GBM) is the most common and devastating primary brain cancer, with an age-adjusted incidence rate of 3.23 per 100,000 population and 12,000 annual average cases, accounting for 57.7% of gliomas and 48.6% of all malignant brain tumors [1]. However, despite extraordinary efforts to improve outcomes, very few therapies have impacted the current standard of care, reflected in median overall survival (OS) of 14.6–16 months and a 5-year survival rate of 4.6% [2,3,4,5,6,7] with optimal treatment patterns.

The current standard therapy includes maximal safe resection and adjuvant chemoradiation [2,5,6]. The use of radiation in patients diagnosed with gliomas is a therapeutic cornerstone that has allowed for further improvement in median OS and quality of life. The first randomized clinical trial, which was performed more than 40 years ago by Walker et al., showed that radiation was able to improve the median OS compared to the control groups (carmustine (BCNU) alone) by 16 weeks, resulting in 34.5 weeks vs. 18.5 weeks [3]. Later, the same group showed similar results and an improvement in survival with use of radiation [8]. Interestingly, the authors also concluded that the use of a nitrosourea could help increase the benefit of radiation. The current treatment paradigm for GBM was established in 2005, when Stupp et al. published the landmark paper showing an improvement in OS with the addition of temozolomide to radiation in a concomitant and adjuvant fashion [2]. Radiation plus temozolomide increased the OS from 12.1 months to 14.6 months when compared to radiation alone in patients with prior surgical resection. Furthermore, the 2-year survival rate also improved with the addition of temozolomide, from 10.4% to 26.5% [2]. Thus, the role for radiotherapy (RT) with temozolomide was established; however, no other agent has been able to further enhance its survival benefit despite a multitude of basic science and clinical studies.

The current therapeutic delivery systems in the treatment of GBM include hormone therapy, immunotherapy, gene therapy, radiation therapy, and the submicron systems (nanoparticles and liposomes) [9]. Any agent administered with therapeutic activity is exposed to biological barriers before reaching its target. In fact, certain properties in the microenvironment act as drawbacks in their interaction: bioavailability, solubility, degradation systems, and unexpected effects such as adjacent healthy tissue involvement. Therefore, the specific delivery of drugs to an organ, tissue, or a type of cell is mandatory. Submicron systems (nanoparticles (NP) and liposomes) serve as a vehicle capable of overcoming existing issues on the proper functioning of the therapeutic agent. Since there are many factors that limit therapeutic options for GBM patients, the use of nanomedicine and targeted approaches can improve the delivery, efficacy, and specificity of current and upcoming theranostics [10].

The main challenges in the treatment of GBM lie in the therapeutic specificity without affecting normal cells/healthy tissue and an environment prone to enhance the therapeutic effect. Additionally, the factors that agents must meet to reach the therapeutic target are as follows: (1) stable maintenance in the blood while reaching the tumor microenvironment; (2) escape from elimination systems such as the reticuloendothelial system and the phagocytic mononuclear system; (3) adequate accumulation in the tumor microenvironment through irregular tumor vasculature; (4) penetrance to the tumor interstitial fluid of the tumor microenvironment with high pressure; (5) reaching the active site and interacting with the target cells exclusively [10].

In this setting, NP have now arisen as potential radiosensitizers that can amplify the radiation-induced damage in GBM cells, directly, by physical mechanisms, or indirectly, by tackling radioresistance pathways. In the following sections we will discuss the basis of NP as well as previous and novel strategies to enhance the synergy between radiation and nanotechnology.

## 2. Nanoparticles

NP refer to nanomaterials with at least one of their external dimensions sized between 1 and 100 nm [11]. This small dimension is associated with a high surface/volume ratio, which confers physical and biological properties different from those of conventional materials. NP can be made from a variety of materials such as lipids, compositing polymers, proteins, metals, or semiconductors. Current nanoparticle platforms for tumors can be classified into three major categories including organic-based NP (e.g., liposomes, dendrimers, polymeric NP, micelles, and solid lipid nanoparticles), inorganic-based NP (e.g., iron oxide NP, gold NP, ceramic NP, semiconductor nanocrystals and carbon nanotubes) and hybrid NP (synthesized from two or more types of nanomaterials). An alternative design of nanoparticles may also contain intrinsic thermal, electrical, optical, or magnetic properties that can be served in imaging or therapeutic purposes [10]. Furthermore, these attributes can be tuned by modifying the size, shape, or surface of NP [12,13].

The possibility of tailoring NP properties according to specific interests makes them an appealing option in nano-oncology, as this opens the door to have them reaching out to specific tissues, cell types or even intracellular compartments for diagnostic or therapeutic purposes [14].

### 2.1. Nanoparticle Targeting

**Figure 1:**
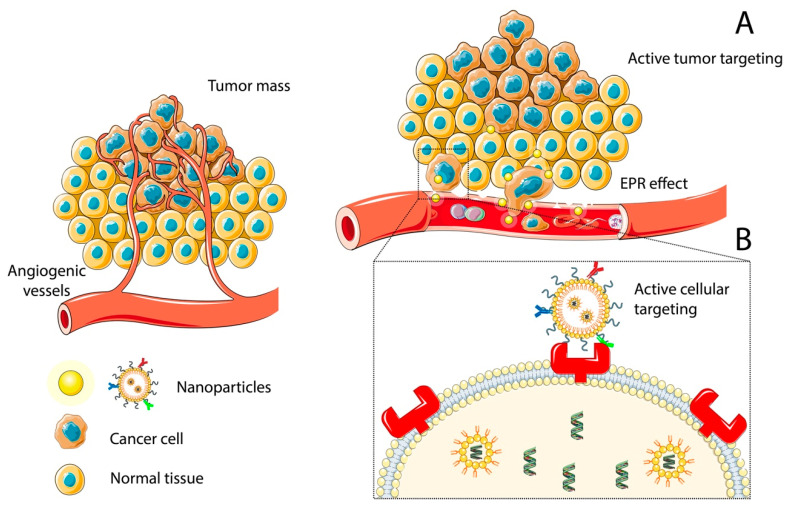
Different types of Nanoparticle Targeting utilized in Glioblastoma Treatment and Diagnosis. (**A**) As passive targeting takes advantage of factors such as nanoparticle size, BBB abnormalities, and EPR effect to reach the tumor tissue by diffusion, active tumor targeting utilizes specific properties of ligands and receptors to increase the interaction between the tumor cell and nanoparticle. (**B**) Active cellular targeting provides nanoparticles the capacity to be selectively engulfed by tumoral cells and localized in specific intracellular spaces, targeting organelles such as the nucleus. Abbreviations: BBB—blood–brain barrier, EPR—enhanced permeability and retention effect.

The process through which a NP interacts with a specific tissue, cell type, or cell compartment in a very selective manner is known as targeting. This can be classified as passive targeting (Figure 1A) or active targeting (Figure 1B), depending on whether this targeting ability is natural or engineered.

Active/passive targeting is the ideal solution to promote NP accumulation in the desired area. As the nucleus is ultimately the final target for many therapeutics, the surface interplay of a drug molecule does not entail necessary its interaction with its subcellular target. The active targeting of tumors can be performed not only by achieving tumor cells but also by localization of the nanocarriers in specific intracellular spaces or organelles, such as nucleus or mitochondria. In addition, active targeting implies reaching the tumor region, targeting the tumor microenvironment, targeting the vascularization of the tumor microenvironment, or directly targeting tumor cells with internalization, simultaneously considering the route of administration [10]. Evidently, active targeting involves the use of carriers bearing various surface ligands to achieve either transport across an intact blood–brain barrier (BBB) or cell uptake following extravasation across a leaky BBB [15,16,17].

Both systems complement each other, increasing accumulation in the tumor region and actively binding to target receptors overexpressed in tumor cell surfaces. Since different delivery methods can benefit from passive/active targeting effects exploiting cellular phenomena in the tumor area, the biological and physicochemical properties of the drug and carrier must be considered.

#### 2.1.1. Passive Targeting: The Role of the Enhanced Permeability and Retention Effect and PEGylation

Passive targeting refers to an inherent mechanism that allows NP to selectively accumulate in the tumor rather than in healthy parenchyma.

In 1986, Matsumura et al. demonstrated that intravenous administration of Evans blue dye accumulated in tumor tissues but not in normal parenchyma, and this difference turned into >5-fold concentration of Evans blue within the tumor when compared to plasma concentrations after 12 h [18]. Importantly, this phenomenon was explained by the fact that Evans blue dye binds to plasma albumin once in the blood stream. A similar phenomenon was observed to happen with the use of radio-labeled plasma proteins such as transferrin (90 kDa) and IgG (160 kDa). On the contrary, smaller proteins were noted not to accumulate within tumors [19,20]. Further studies demonstrated that macromolecules with a molecular weight above the renal thresholds (40 kDa) might accumulate in cancerous tissues, as described before, caused by poor renal filtration and the existence of an abnormal and more permeable neoplastic vasculature. Maeda et al. also showed that when Evans blue dye was directly injected into the tumor, it was retained longer than when injected into healthy tissue, which was hypothesized to be related to an impaired functionality of the lymphatic drainage in the tumor [18].

The phenomenon described above is known as the enhanced permeability and retention (EPR) effect, and relies on the pathophysiological characteristics of tumor vessels, which are more permeable than healthy blood vessels [21,22]. This phenomenon also depends on the characteristics of the particle that is expected to reach the tumor. Thus, the EPR effect allows for the movement of NP with a sizable dimension into the tumor microenvironment, but not through healthy vessels, which are better sealed and prevent this movement [19,20]. This is possible given that NP with diameters of at least 5–10 nm have reduced kidney excretion different from conventional chemotherapeutics. They are usually <1 kDa and filter more easily through the kidney [23]. Consequently, these particles have enough time to move from the bloodstream into the tumor tissue.

Another important factor determining the accumulation of NP within the tumor is the circulation time, as a longer blood half-life will increase the likelihood of NP concentration within the tumor tissue. Additionally, coating NP with polymers such as poly(ethylene glycol) (PEG) (also known as PEGylation) represents an strategy to increase the circulation time, which is highly valuable for passive targeting [24].

PEGylation has an important effect on NP biodistribution, stabilization, and structure in vitro and in vivo [24,25,26,27]. PEG modification improves the circulation time by preventing NP engulfment by the mononuclear phagocyte system (MPS) and subsequent removal from circulation [28,29]. After PEG molecules bind the NP surface, they form a well hydrated inert hydrophilic layer that prevents the binding of other molecules via steric repulsion forces [30]. Thus, PEGylation increases NP stability in biological fluids with high-salt concentrations, such as the blood, where PEG help circumvent non-specific cell–protein interactions, such as those mediating opsonization, which could foster NP recognition and phagocytosis by MPS [28,30].

Overall, despite the role EPR effect and PEGylation on the distribution of NP within brain tumors, the BBB is still very restrictive. Given its cellular composition, tight junctions connecting endothelial cells and efflux pumps prevent molecules from penetrating the BBB [31]. Therefore, physicochemical properties, such as molecular weight, lipophilicity, and charge of targeted therapeutic molecules, are arduously studied [32].

Breakdown of the BBB is common in high-grade gliomas and brain metastases, defining the blood–brain tumor barrier (BBTB) [33]. The dysfunctional barrier grants a hyperpermeability property to the endothelium. Considering this state, drugs relying on EPR effect for delivery could be exploited for targeting macromolecules of the tumor.

Hence, other strategies are necessary to improve NP distribution more selectively. Active targeting is one of these strategies and will be discussed below.

#### 2.1.2. Active Targeting

Active targeting refers to the process that allows NP to selectively distribute into specific cell types or even intracellular compartments (Figure 1B) [23]. This involves the design of specific moieties for NP biofunctionalization. Active targeting can be achieved by different methods.

One of these methods is known as ligand targeting, this involves the selection and designing of specific ligands which will bind to the NP surface (biofunctionalization) to mediate the precise interaction between NP and the desired target cell [23]. This interaction between the ligand and the cell happen through cellular receptors/membrane proteins that may or may not lead to cellular internalization [34]. In general, these ligands can be classified as antibody or non-antibody ligands, each of these with pros and cons. Monoclonal antibodies (mAbs) or mAbs fragments have a high degree of specificity for the target tissue and a potentially regulable binding affinity [35,36,37]. An additional advantage derived from the use of mAbs is the possibility of synergy between the engulfed NP and the downstream antibody signaling [34,38]. The non-antibody ligands include molecules that bind to specific cell receptors, and in the case of GBM these ligands can be folic acid, hyaluronic acid, arginyl-glycyl-aspartic tripeptide (RGD) peptide, epidermal growth factor (EGF), and others [39]. However, although these non-antibody ligands enhance target specificity, they can still bind some non-target tissues as well as compete for the target receptor with free ligands, such as folate, that can also come from diet and reach high levels in plasma [34]. Any of the previously mentioned ligands can be directly or indirectly (i.e., after PEGylation) linked to the NP, and thus target cancer cells, tumor stromal cells, or tumor blood vessels [23].

### 2.2. Nanoparticle Radiosensitization Efforts

RT remains the primary treatment modality for unresectable GBM [40,41]. However, there are several drawbacks associated with this approach, including radioresistance due to tumor hypoxia, limitations in radiation dose due to normal tissue constraints, especially in the setting of previous radiation in recurrent tumors, and the lack of target specificity [42,43,44]. Therefore, it is clear that new approaches to improve the efficacy of radiation treatment for patients diagnosed with glioma are necessary.

Different approaches have been proposed to overcome these roadblocks, broadly falling into two main categories: (1) implementation of advanced RT techniques and, (2) development of a new generation of treatment agents that sensitize cells to ionizing radiation (radiosensitizers) by improving their relative biological effectiveness (RBE) [45]. Thus, the use of radiosensitizers is a potential approach to improve RT efficacy.

In this setting, NP have been tested as radiosensitizing agents as well as carriers of other radiosensitizing agents, with studies demonstrating promising results after photon and particle radiation [44,46,47]. This strategy using nanomedicine aims to load NP with drugs that sensitize cancer cells to radiation, and thereby enhance the killing of residual disease and prevent treatment resistance [48]. NP as radiosensitizers has been extensively explored over the past decades; but recently high atomic number NP, such as gadolinium (Z = 79), hafnium (Z = 72), platinum (Z = 78), gold (Z = 79) or bismuth (Z = 82) have been investigated [49]. The rationale relies on the amplification of radiation effects when activated by photons of keV to MeV energies, electrons, neutrons, or fast ions (>50 MeV amu^−1^) [50,51].

Several studies have been designed to introduce NP as potential enhancers of RT efficacy. Gold nanoparticles (AuNPs) are an important category among the radiosensitizers due to their high stability, biocompatibility, high atomic number, and the ability to be synthesized at diverse sizes and characterizations [52]. Extensive preclinical studies have demonstrated significant local enhancement of the absorbed dose using AuNPs compared to kilovoltage (kV) X-rays alone. The impact of these structures is attributed to the photoelectric effect, resulting in a high mass energy coefficient relative to soft tissue [44,53]. Kazmi et al. evaluated the radiosensitization effect on U87 GBM cells in the presence of 42 nm AuNPs and irradiated with clinical 6 MV photon beam. They showed a significant improvement in radiosensitivity using AuNPs. Similarly, there was a significant difference in the surviving fraction between the GBM alone group and the GBM plus AuNPs group. With this design, Kazmi et al. demonstrated that U87 cells irradiated at 2 Gy using a clinical platform enhanced radiosensitization by acting in synergy with AuNPs [44]. Moreover, Kunoh et al. initially examined the cytotoxicity of DNA-generated AuNPs in human U251MG-P1 cells that had cancer stem cells’ properties. Then, they studied the radiation sensitivity of the cells associated or unassociated with AuNPs. This group demonstrated that pretreatment of the cells with AuNPs abrogated the radioresistance of cancer cells [54]. Additionally, it did not induce apoptosis in the cells but enhanced the number of abnormal nuclei, causing cell death by mitotic catastrophe. Comparably, Hua et al. developed a novel radiosensitizing agent-containing NP to enhance the radiation effect on gliomas. A metronidazole-based hydrophobic core was synthesized, mixed with angiopep-2 and lecithin, to self-assemble by nanoprecipitation. Within this core, doxorubicin (DOX) was encapsulated, and its contents released under hypoxic conditions. The nitro groups of metronidazole are converted to hydrophilic amino groups, which destabilize the NP and promote the release of DOX. This effect was assessed in both in vivo and in vitro models. Hua et al. demonstrated that these particles could deliver hydrophobic chemotherapy, achieving the effect of concurrent chemotherapy and radiation and inducing the release of hydrophobic chemotherapeutics under hypoxic conditions. Additionally, the in vitro and in vivo results demonstrated significant inhibition of glioma growth and efficient targeting of gliomas [40].

Intending to limit toxicity to surrounding healthy tissue and enhance radiation damage, different modes of locoregional drug delivery, such as stereotactic radiosurgery (SRS), are being developed [55]. Séhédic et al. investigated the in vivo cellular targeting of rhenium-loaded lipid nanocapsules (LNC) in a locoregional strategy. In this study, the impact of the mutual action of intracerebral internal vectorized RT and active CXCR4-immunotargeting was investigated using 12G5-conjugated LNC^188^Re in a xenogeneic and orthotopic glioma model of human U87MG cells expressing the CXCR4 receptor implanted in Scid mice. CXCR4 is blockaded by function-blocking antibody 12G5, which is notably reported to inhibit SDF-1-induced GBM cell proliferation [55,56]. This group demonstrated that a 12G5-LNC^188^Re single infusion delivered by convection-enhanced delivery (CED) resulted in significantly improved median survival that was accompanied by locoregional effects on tumor development, including hypovascularization. Their findings support the hypothesis that the use and optimization of intracerebral active targeting of nanocarriers loaded with radiopharmaceuticals may lead to considerable benefits in human trials.

**Figure 2:**
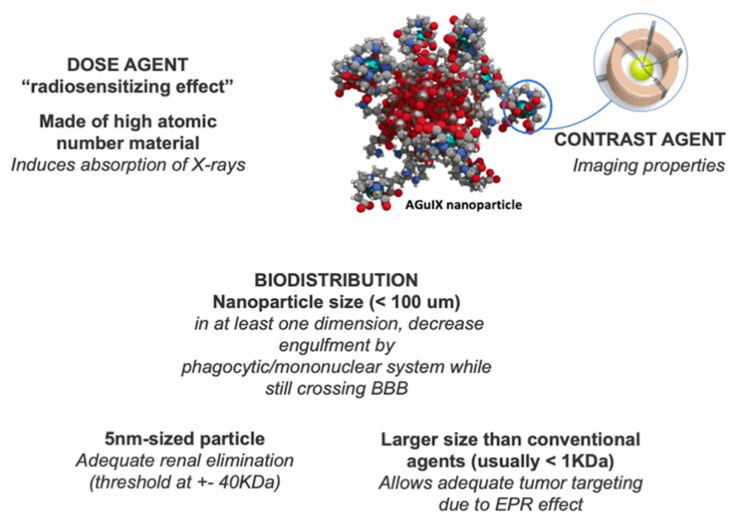
Features of an Adequate Theragnostic Nanoparticle for Glioblastoma. Description of desirable features in a theragnostic nanoparticle, most of them are related to size and nature of nanoparticle. Imaging provided by Dr. Loeffler *with permission* (ref [39,57,60]).

Image-guided RT presents a unique area of overlap with nanomedicine, specifically when using MRI-guided therapy with gadolinium. Gadolinum, with its high atomic number, allows for improved delineation of the tumor [57]. The strategy to enhance the imaging contrast and improve theranostic applications of chelating species includes coupling these agents to the surface of NP or incorporating them into nanostructures [58,59]. Le Duc et al. developed a family of ultrasmall gadolinium-based NP for MRI visualization and radiosensitization called AGuIX (Activation Guided by Irradiation by X-rays), to compare their efficiency for magnetic resonance imaging and radiosensitization to those of the commercial gadolinium based molecular agent. Two main types of NP were proposed with similar physico-chemical properties, radiosensitizing properties, and in vivo biodistribution profiles: polysiloxane core coated with DTPA (Diethylene Triamine Pentaacetic Acid) and molecules incorporating DOTAREM, a commercial gadolinium-based molecular agent [57]. They established a protocol consisting of microbeam RT 20 min after the injection of a specific quantity of gadolinium. The results show an increase in survival time to 102.5 days with AGuIX particles; this radiosensitizing effect could be explained by the persistent tumor uptake of the particles, inducing a significant nanoscale dose deposition under irradiation (Figure 2) [57].

Despite these promising initial results, the evidence for its clinical application is very limited. Currently, the gadolinium-based AGuIX was included in a phase I study protocol called NANO-RAD (NCT02820454) [61,62]. As AGuIX functions as a magnetic resonance imaging contrast and a radiosensitizer, the study aimed to investigate the safety and maximum tolerated dose of systemic administration in combination with whole-brain radiotherapy (WBRT) in patients with multiple brain metastases not suitable for stereotactic RT. Additionally, an MRI protocol was established to evaluate drug distribution in brain metastases and surrounding healthy tissues. Patients received WBRT of 30 Gy in 10 daily sessions over 2 weeks; AGuIX was administered intravenously 4 h before the first WBRT session. Patients with measurable brain metastases received escalating doses of AGuIX nanoparticles (15, 30, 50, 75, or 100 mg/kg intravenously) on the day of initiation of WBRT (30 Gy in 10 fractions) in 5 cohorts of 3 patients each. The results show that of 15 patients with 354 metastases, no dose-limiting toxic effects were observed up to AGuIX 100 mg/kg. Thirteen of 14 evaluable patients had stabilization or reduction of tumor volume. NP concentration after administration was proportional to the injected dose. MRI showed a correlation between contrast enhancement and tumor response. Thus, combining AGuIX with RT showed safety and tumor response, targeting brain metastases, and remaining within tumors for up to 1 week.

In this regard, CED of OS2966, an anti-CD29 (Beta1 integrin) monoclonal antibody, was included in a phase 1 study to determine if this drug, when delivered directly to the brain of adult patients with recurrent/progressive high-grade glioma (HGG), is safe and well tolerated [63]. This study is an open-label, dose-escalation, two-part study. Tumor tissue will be collected in both study parts to evaluate how well OS2966 targets malignant cells and to confirm the mechanism of action. All enrolled patients will also receive standard supportive care therapy. The lack of progress in treating malignant brain tumors is because of the complex environment where tumor growth occurs. Moreover, their radioresistance compared to the brain and the poor distribution of cytotoxic agents are important factors worth exploring by coupling NP as new radiosensitizers with additional fields to maximize its benefits.

Current efforts, either in preclinical models or ongoing clinical trials in the treatment of GBM, are summarized in Table 1.

## 3. Delivery Methods for Nanoparticles in Malignant Gliomas

**Figure 3:**
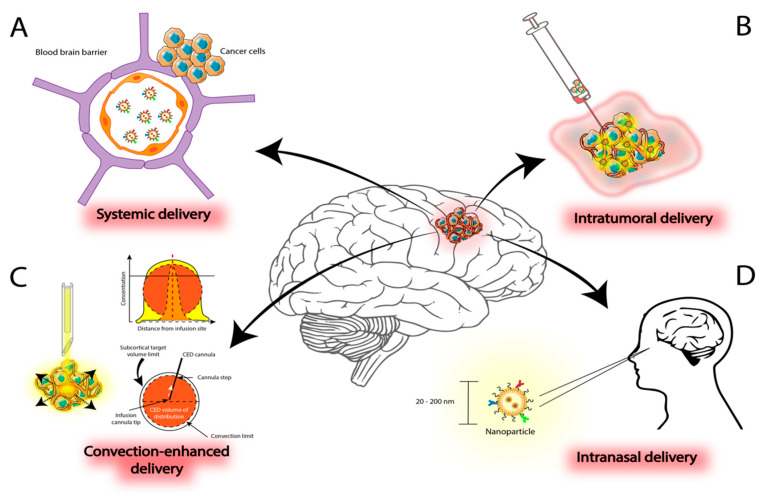
Different routes of Distribution of Nanoparticles in Glioblastoma. (**A**) Systemic delivery is a common route of distribution; however, it may be limited by the BBB. (**B**) Intratumoral delivery overcomes limitations imposed by BBB constraints but the NP may be localized to the site of injection. This limitation is improved by CED (**C**). (**D**) Intranasal delivery is a promising route for NP delivery, but it is still under study. Abbreviations: BBB—blood–brain barrier, NP—nanoparticle, CED—Convection-enhanced delivery.

Delivery of therapeutics to the brain is an ongoing challenge in the treatment of brain tumors, particularly in GBM. Despite efforts to devise new treatment strategies, these lesions invariably recur as more aggressive and resistant to conventional treatment. Different approaches have been tested for NP administration in clinical and pre-clinical settings as nanotechnology rises as an alternative in the armamentarium to treat this devastating disease (Figure 3). Nanomedicine approaches can open therapeutic opportunities to improve effectiveness and outcomes through synergistic effort with established therapies.

### 3.1. Systemic Administration

Intravenous administration has been the most widely used route of administration for different types of NP tested preclinically. Kefayat et al. investigated folic acid (FA) and BSA decorated gold nanoclusters (FA-AuNCs) as a radiosensitizer in tumors derived from C6 cells (rat glioma). To evaluate FA-AuNCs intracranial distribution, the nanoclusters were injected intravenously in tumor-bearing rats one week after intracranial implantation of glioma cells. Biofunctionalization with FA helped to achieve a higher concentration of AuNCs in cancer tissue compared to healthy brain parenchyma. This also caused significant deposition of radiation beams energy at the tumor and consequent cancer cells damage and enhancement of RT efficacy [52]. Groups receiving radiation were delivered a single dose of 6 Gy using a compact linear accelerator. Subsequently, Kefayat et al. demonstrated that glioma-bearing rats’ median survival times were significantly higher at RT + FA-AuNCs (24.5 days) compared with radiation alone (18 days) [52].

Since heavy elements in NP were proposed as an appropriate alternative to improve radiosensitization, Bhattarai et al. studied gold nanotriangles (AuNTs) as a possible radiation sensitizer in mice. To produce high quality monodisperse AuNTs, they used a convergent batchwise seed-mediated method. In vitro and in vivo tests were performed to determine effectiveness: uptake of AuNTs with different molecular weights ligands, cytotoxic dose in MCF-7, radiation experiments with radio-resistant U87MG human GBM cells and biodistribution and radiation effects in mice with U87MG implanted tumors. After tumor implanted growth, AuNTs were injected intravenous in bearing mice. As concluded, AuNTs have no cytotoxicity and exhibit good performance in radiosensitization [64].

Temozolomide (TMZ), a prodrug releasing a DNA alkylating agent, is considered as an effective drug in patients with GBM due to its penetrating BBB capacity. Despite being generally well tolerated, toxicities associated with TMZ include nausea, fatigue, and hematological side effects, including thrombocytopenia [60,69,70]. Zong et al. developed angiopep-2 (A2) modified lipid-poly NP for TMZ delivery to achieve synergistic effects against glioma. The NP were injected in tail vein to subsequently penetrate the BBB and enter the glioma tumor due to the EPR effect and active target [71]. In this study, mice with xenograft glioma were divided into seven groups (*n* = 10). Different lines of treatment were evaluated: PBS, PBS + RT, A2-PLGA + RT, free TMZ + RT, A2-P(MIs)25 + RT, A2-P(MIs)25/TMZ + RT and A2-PLGA/TMZ. With this design, the in vitro and in vivo results demonstrated that these A2- P(MIs)25/TMZ can efficiently target glioma to increase the concentration of TMZ (hypoxic cell radiosensitizers). Additionally, the therapeutic studies showed that A2-P(MIs)25/TMZ can effectively inhibit the growth of glioma cells and significantly improve mice survival time without adverse effects [71].

Additionally, intravenous administration requires attention in certain aspects, especially a detailed biodistribution analysis, to identify the mechanisms of particle elimination and discriminate between the particle concentration reached in the tumor and in the surrounding healthy tissue [45]. Likewise, other studies have been carried out via intravenous injection using high-Z metal NP for enhanced RT in preclinical models [72,73,74,75,76].

### 3.2. Intratumoral Delivery

The intratumoral approach, an alternative route of administering NP, is designed to overcome problems associated with drug delivery, including dispersion, penetration, and retention of the agent. Improving absorption and diffusion of the agent itself within the tumor microenvironment would result in better outcomes and improved efficacy of drug administration.

In this regard, Peidang et al. evaluated the efficacy of intratumoral administration of silver nanoparticles (AgNPs) in combination with a single dose of ionizing radiation at clinically relevant megavoltage energies for the treatment of C6 glioma-bearing rats [77]. NP were stereotactically administered on day 8 after tumor implantation. Rats bearing glioma received 10 Gy radiation one day following AgNPs injection. The antiproliferative and proapoptotic effects were obtained when gliomas were treated with AgNPs followed by RT. The therapeutic efficacy of AgNPs in combination with radiation in the absence of systemic toxicity is encouraging for further application in clinical settings. Later, Peidang et al. compared the radiosensitizing effects of AuNPs and AgNPs on gliomas at clinically relevant megavoltage energies [65]. Both high-Z metal NP potentiated the in vitro and in vivo antiglioma effects of radiation, although there were higher rates of apoptotic cell death using AgNPs. Additionally, when radiation was added, AgNPs showed a significant increase in autophagy levels as compared with AuNPs. These findings support the potential application of AgNPs as an effective nanoradiosensitizer for the treatment of glioma with superior outcomes.

To increase specific and long-term retention of NP, Brachi et al. studied the system NP-Hydrogel [78]. The particles were embedded within a thermosensitive hydrogel, following intratumoral administration in GBM. Using a fluorophore (BODIPY) encapsulated inside infrared dye (cyanine 7)-labeled polyurethane (PUR) NP, retention and distribution dynamics were subsequently examined in orthotopic GBM-bearing mice over time. The results show that NP-Hydrogel had a significantly longer intratumoral retention compared to free NP and covered a significantly larger area of the tumor and the peritumor region. Additionally, these findings suggest that combining both delivery systems may increase the therapeutic window of intratumoral-administered drugs. This encourages combining delivery methods and approaches to enhance the therapeutic effects of drugs used in the treatment of brain tumors in clinical settings.

## 4. Novel Strategies to Synergize with Nanoparticle-Based Radiosensitizers

### 4.1. Stem Cells

Mesenchymal stem cells (MSCs) possess certain qualities that make them suitable for this strategy, such as hypoimmunogenicity, fast ex vivo expansion, and inherent tumor-tropic and migratory properties [79]. These cells can track infiltrating cancer cells, which are responsible for promoting future recurrences, even beyond the location of the macroscopic tumor itself. Bioengineering techniques have been developed to modify these cells for inoculating various cell therapy alternatives, such as viral vectors, small interfering RNA (siRNA), prodrugs, and NP. Certainly, combinatorial approaches are currently of great interest given the considerable enhancement of therapeutic effects. Among them is the inhibition of glioma growth through controlling aspects involved in the cell cycle, and improving NP-related properties, such as improving diffusion toward the tumor nucleus and distal infiltrating tumor foci. This implies the internalization of the content in a stem cell before migrating and thus taking advantage of the cellular tropism, or simply the interaction of surfaces as conjugation for the same purpose. Therefore, it is considered a dual advancement. Our group has previously described the potential advantages of using combined approaches such as nanoparticle-based stem cell therapy in malignant glioma: (1) stem cells carry bigger cargos, increasing NP loading capacity; (2) big NP can be transported through the BBB; (3) stem cells improve delivery localizing therapeutic effect into the hypoxic central glioma core, overcoming treatment-resistant cancer stem cells (CSC); (4) tracking while delivering their cargo to CSC leaving the tumor bulk, which is considered to be one of the main reasons for tumor recurrence [23].

### 4.2. Stereotactic Radiosurgery

An additional potential approach to synergize with radiosensitizers is the use of SRS rather than standard fractionated RT in GBM patients. In general, RT of the brain involves two main modalities of radiation delivery: fractionated RT, and SRS. Radiosurgery has arisen as an important clinical tool in the management of brain tumors, as it can spare much more healthy brain tissue when compared to standard fractionated RT.

#### 4.2.1. Potential Benefit of Radiosurgery in Glioblastoma

The current management of GBM includes maximal safe resection followed by adjuvant chemoradiation. This standard treatment implies the delivery of traditionally fractionated RT, where 60 Gy are delivered over six weeks to a volume including postsurgical cavity plus 2 cm margin. On the other hand, SRS delivers a large dose of highly conformal radiation to a specified target while limiting the dose to normal tissue in a single or hypofractionated intervention (up to five fractions). SRS has been studied in preclinical models of glioma. SRS induces cellular changes within the tumor, as well as modifying the tumor microenvironment. Briefly, the high dose delivered by SRS can ablate dividing cells, inducing senescence within non-ablated cells, stimulate local tumor immunity, and promote antitumor immune responses via a host of molecular mechanisms [80]. In addition, SRS causes the release of tumor-associated antigens, which initiates the cascade of an adaptive immune response, activating antigen-presenting cells and further priming of cytotoxic CD8^+^ T cells [81].

Taken together, radiation might counteract the immunosuppressive tumor microenvironment of GBM by increasing major histocompatibility complex (MHC) class I expression [82], enhancing the presentation of normally suppressed tumor-associated antigens while increasing the expression of proinflammatory cytokines, promoting dendritic cell maturation, and downregulating Fas ligand expression [83,84,85]. Immunogenic cell death (ICD) is characterized by the exposure of calreticulin on the surface of dying cells, the release of ATP, and the secretion of high-mobility group box 1 protein into the tumor microenvironment. These markers of ICD can expand antigen presentation and subsequent CD8^+^ T cell activation in GBM [82,86]. In this context, Zeng et al. investigated the combination of anti-PD-1 immunotherapy with stereotactic radiation in a mouse orthotopic GBM model, on the premise that immunotherapy can work synergistically with radiation, which has been shown to increase antigen presentation and promote a proinflammatory tumor microenvironment, as noted above. This group demonstrated that combining anti-PD-1 therapy plus radiation improved survival compared with standard modality alone. Although neither PD-1 blockade nor single-session focal RT alone eradicated intracranial gliomas, the combination of these therapies generated robust, durable responses [83]. Despite efforts to increase the effectiveness of therapeutics in the tumor microenvironment, a major challenge for this approach is reaching tumor cells that are resistant to these modalities and do not respond to therapy. Therefore, it is of interest to study other therapies that can synergize and increase effectiveness, to overcome the heterogeneous and malignant behavior of tumor cells. Radiosensitization is one of these approaches and will be discussed in the following subsection.

In the last decade, neoadjuvant SRS was proposed as a therapeutic approach [87,88,89]. Routman et al. described the role of preoperative SRS in brain metastases. When compared to postoperative SRS, preoperative SRS could offer comparable local control, decreased radiation necrosis rates and leptomeningeal disease, and would avoid the delay introduced by the required time for wound healing between surgery and postoperative radiation. The lack of pathologic confirmation before preoperative SRS is a concern, although less so given more reliable non-invasive diagnostic methods, such as liquid biopsies, that allow clinicians to more accurately determine malignancy without the need for biopsy [87]. Although SRS has been studied in GBM, the role of preoperative SRS in this pathology is still not clear [90].

#### 4.2.2. Nanoparticle Radiosensitization and Preoperative Radiation in GBM

With recent discoveries, it has become apparent that improving the current approach to overcome the barriers in the treatment of GBM could potentially improve long-term outcomes in patients. The cytotoxicity of RT is caused by DNA double-strand breaks (DSBs) generated directly by the radiation or indirectly through the generation of reactive oxygen species, as noted above. Nevertheless, only 2% of the damage caused by this technique generates these cytotoxic DSBs directly [91]. Therefore, other strategies are required to increase tumor toxicity caused by RT to improve effectiveness and obtain better long-term results. SRS is safe and is routinely used to deliver RT to intracranial tumors. Although the infiltrative nature of GBM would preclude the use of SRS as a primary treatment modality, new data suggest that SRS should be reexamined when used in combination with different therapeutics such as NP [83]. This combination could enhance the response of tumor cells by inhibiting resistance properties, improve radiation dosimetry, or improve the delivery of chemotherapy. Additionally, timing plays an important role when radiation is employed. A neoadjuvant approach to RT might offer potential improvements in local control rates while decreasing toxicity. Thus, preoperative SRS could be used in conjunction with radiosensitizers. To date, high atomic element nanomaterials are often utilized as radiosensitizers due to their unique photoelectric decay characteristics. AuNPs are widely investigated and are considered as ideal radiosensitizers for RT because of their X-ray absorption and physical properties. Despite promising results, its clinical translation still faces many challenges. Among the few advances in the clinical use of preoperative RT and NP, NBTXR3 NP were investigated in a phase 1 trial (NCT01433068) that started in 2011 and was completed in 2015. The results show that human injection (22 sarcoma patients in France) was well tolerated until 10% of tumor volume was achieved. NP were delivered alongside preoperative external beam RT and this did not result in leakage of these NP into the adjoining healthy tissues [92]. Alternative strategies have been proposed to balance treatment outcomes and side effects; however, to our knowledge, clinical studies designed to investigate the more suitable time for applying RT plus NP have not yet been performed in GBM.

In this context, more research is needed with alternative approaches to overcome the therapeutic resistance found during glioma therapy; opting for joint efforts of technologies to enhance effects. Clinical studies using stem cells as therapeutic tools in glioma patients show encouraging results. As our group has previously described, MSCs derived from adipose tissue are affordable and malleable enough to characterize with joint nanotechnology [23,39]. In addition, this approach can be synergized with of other technologies, such as SRS, and ideally maximize the therapeutic value ratio for patients with GBM.

Radiation and surgical resection are the keystones in the treatment of GBM. However, it is mandatory to involve other technologies to achieve better control of the disease and become current therapies more effective. Precisely, current challenges limiting the study of radiosensitizers in clinical trials lie in three fundamental categories: drug interactions, delivery systems, and preclinical models. To continue working towards this approach, we present the current landscape, summarized in Table 2.

With a multidisciplinary effort, radiosensitizers could become a standard of treatment in the GBM approach. It is promising based on the results obtained so far and may help address the therapeutic deficiencies of current GBM management.

## 5. Conclusions

The use of NP for radiosensitization in GBM is an appealing approach that deserves thoughtful consideration. NP offer several therapeutic advantages derived from the possibility of bioengineering them to fit tumor-specific therapeutic requirements. Despite substantial efforts, this approach has not yet impacted the care of GBM patients, although synergistic approaches may boost its clinical significance.

One of these approaches is in combination with radiation, which can be delivered in a traditional or novel approach. We expect that nanomedicine and preoperative SRS will find their way together in radiosensitization without excluding additional potential synergistic proposals, such as simultaneous modulation of the tumor microenvironment.

## Figures and Tables

**Table 1:** List of current radiosensitizers used in clinical and preclinical models.

Agent	Study Type	Treatment Regimen
Gold nanoparticles (NP)	Preclinical	Gold NP + clinical 6 MV (megavoltage) photon beam in U87 glioblastoma cells [44]
Gold NP	Preclinical	X-ray irradiation + Gold NP in U251MG glioblastoma cells [54]
Folic acid decorated gold nanoclusters	Preclinical	Gold nanoclusters + 6 Gy radiation dose in C6 rat glioma cells [52]
Gold nanotriangles	Preclinical	Gold nanotriangles + 250 kVp X-rays in U87MG human glioblastoma cells [64]
Silver NP	Preclinical	Silver NP + 6 MV X-rays beams with linear accelerator in U251 glioblastoma cells [65]
Silver NP	Preclinical	Silver NP + 6 MV X-rays beams with linear accelerator in U251 glioblastoma cells and C6 glioma cells [66]
Graphene oxide NP as carrier of IUdR	Preclinical	Graphene NP + 8 Gy radiation in C6 glioma cells [67]
Gadolinium-based AGuIX NP	Phase 1 clinical trial	AGuIX NP injected IV + WBRT^3^ (30 Gy/10 fractions) [68]
RRx-001	Phase 1 clinical trial (ongoing)—NCT02871843	RRx-001 + TMZ (temozolomide) + radiation
Sulfasalazine	Phase 1 clinical trial (ongoing)—NCT04205357	Sulfasalazine + stereotactic radiotherapy
NVX-108	Phase 2 clinical trial (ongoing)—NCT03862430	NVX-108 + TMZ + radiotherapy
Carboplatin	Phase 1 & 2 clinical trial (ongoing)—NCT03672721	Carboplatin + radiotherapy
Chloroquine	Phase 2 clinical trial (ongoing)—NCT02432417	Chloroquine + TMZ + radiotherapy
Adavosertib	Phase 1 clinical trial (ongoing)—NCT01849146	Adavosertib + TMZ + radiotherapy
AZD1390	Phase 1 clinical trial (ongoing)—NCT03423628	AZD1390 + radiotherapy

**Table 2:** Current challenges and future directions of radiosensitization.

Problem	Main Challenges Ahead
Drug interactions	Experimental studies focused on improving radiosensitization of therapeutical agents and replication of antineoplastic effect within the microenvironment.
Delivery systems	Delivering agents to the tumor site more precisely, leveraging existing technologies to monitor response and effectiveness (e.g., post-treatment markers, advanced imaging techniques).Focus studies to assess the functioning of delivery systems and their efficacy against biological barriers, such as bypassing systemic metabolism and the blood–brain barrier.
Preclinical models	Adapting preclinical models of simulated tumor microenvironment for radiosensitizer assessment to produce more accurate tumor response data.
